# Aortic Root Abscess Causing Atrioventricular Block in a Patient With Infective Endocarditis: A Fatal Case

**DOI:** 10.7759/cureus.90807

**Published:** 2025-08-23

**Authors:** Taha Khalid, Ammad Naeem

**Affiliations:** 1 Department of Internal Medicine/Hospital Medicine, St. Mary's Medical Center, Huntington, USA; 2 Department of Internal Medicine, Charleston Area Medical Center, Charleston, USA

**Keywords:** aortic root abscess, cardiac conduction abnormalities, conduction disturbance, heart block, infective endocarditis

## Abstract

Aortic root abscess is a rare but serious complication of infective endocarditis (IE), which can lead to conduction abnormalities such as atrioventricular (AV) block. We describe a case of a 48-year-old man with end-stage renal disease on hemodialysis who presented with weakness and recurrent falls, later found to have methicillin-sensitive Staphylococcus aureus bacteremia originating from a dialysis catheter. Despite appropriate antibiotic therapy and catheter removal, the patient developed a new first-degree AV block, which progressed to a high-grade block. Transesophageal echocardiography revealed a large vegetation at the base of the aortic valve with aneurysmal calcifications and suspected aortic root abscess, later confirmed on cardiac MRI. Due to significant comorbidities, he was not a surgical candidate and ultimately deteriorated into shock and died despite long-term antibiotic treatment. This case underscores the need for high clinical suspicion for intracardiac abscesses in patients with IE and new conduction disturbances, as early diagnosis and surgical intervention are critical to improving outcomes.

## Introduction

Infective endocarditis (IE) remains a challenging clinical condition with a broad spectrum of presentations and complications. Despite advances in antimicrobial therapy and diagnostics, the morbidity and mortality associated with IE remain high, particularly in patients with comorbid conditions such as end-stage renal disease (ESRD) [1-3]. Among the most severe complications is perivalvular extension of infection, including the formation of intracardiac abscesses, which occur in approximately 30-40% of cases. These abscesses often involve the aortic root and are associated with high mortality rates, especially when not promptly identified and treated [4-6].

Intracardiac abscesses can lead to disruption of the cardiac conduction system due to their proximity to the atrioventricular (AV) node and the His-Purkinje system, particularly when they occur near the aortic valve annulus. This can manifest as new-onset conduction abnormalities such as PR prolongation, bundle branch blocks, or advanced AV block [1,7,8]. The development of new conduction delays in a patient with IE should prompt urgent evaluation for a potential abscess using imaging modalities such as transesophageal echocardiography or cardiac MRI [7,9]. 

Patients with IE on hemodialysis are at increased risk for bloodstream infections, often with Staphylococcus aureus, and have a greater likelihood of complications such as endocarditis and abscess formation due to vascular access devices [6,10]. Surgical intervention remains the cornerstone of treatment for aortic root abscess; however, high operative risk may preclude surgery, leaving limited options and a poor prognosis [4,5]. 

In this report, we present the case of a dialysis-dependent patient who developed an aortic root abscess complicated by second-degree heart block and fatal hemodynamic collapse, underscoring the importance of timely diagnosis and the limitations of conservative management in high-risk individuals.

## Case presentation

A 48-year-old African American man with a past medical history of ESRD on hemodialysis, coronary artery disease, hypertension, and anemia presented to the emergency department with complaints of progressive weakness and recurrent falls over the past three days. He also reported subjective fevers and chills but denied chest pain, shortness of breath, palpitations, or focal neurological deficits.

On initial examination, the patient was febrile to 38.9°C, hypotensive (90/60 mmHg), and tachycardic (heart rate 108 bpm). Physical examination was notable for mild pallor and petechiae near the tunneled dialysis catheter insertion site; the catheter site showed no signs of local inflammation. Cardiopulmonary and neurologic exams were unremarkable. No heart murmurs, peripheral stigmata of infective endocarditis, or signs of volume overload were appreciated.

Laboratory evaluation on admission, as shown in Table 1, revealed abnormalities suggestive of systemic inflammation, anemia, and thrombocytopenia. Inflammatory markers were significantly elevated. Liver enzymes were mildly elevated, with signs of hypoalbuminemia. Blood cultures were positive for methicillin-sensitive Staphylococcus aureus (MSSA). A transthoracic echocardiogram showed an echodensity on the tricuspid valve without significant regurgitation or vegetation. The electrocardiogram (ECG) initially showed a normal sinus rhythm. 

**Table 1:** Admission laboratory values with interpretation and reference ranges. MSSA: Methicillin-sensitive Staphylococcus aureus

Parameter	Value on Admission	Reference Range	Interpretation
White Blood Cell Count	15.8 ×10⁹/L	4.0 – 11.0 ×10⁹/L	High
Hemoglobin	8.9 g/dL	13.5 – 17.5 g/dL	Low
Platelet Count	65 ×10⁹/L	150 – 450 ×10⁹/L	Low
C-Reactive Protein (CRP)	33	<5 mg/L	High
Erythrocyte Sedimentation Rate (ESR)	67	<20 mm/hr	High
AST/ALT	77/167	AST: <40 U/L, ALT: <56 U/L	High
Albumin	2.0	3.5 – 5.0 g/dL	Low
Blood Cultures	MSSA positive	Negative	Positive

**Figure 1:**
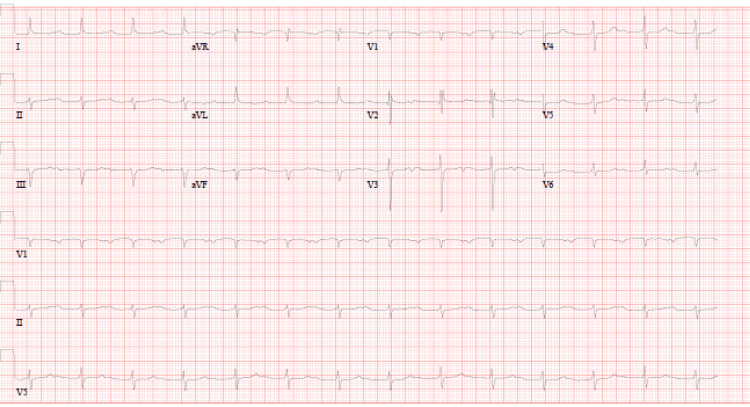
EKG showing prolonged PR interval indicating first-degree AV block AV: Atrioventricular

**Figure 2:**
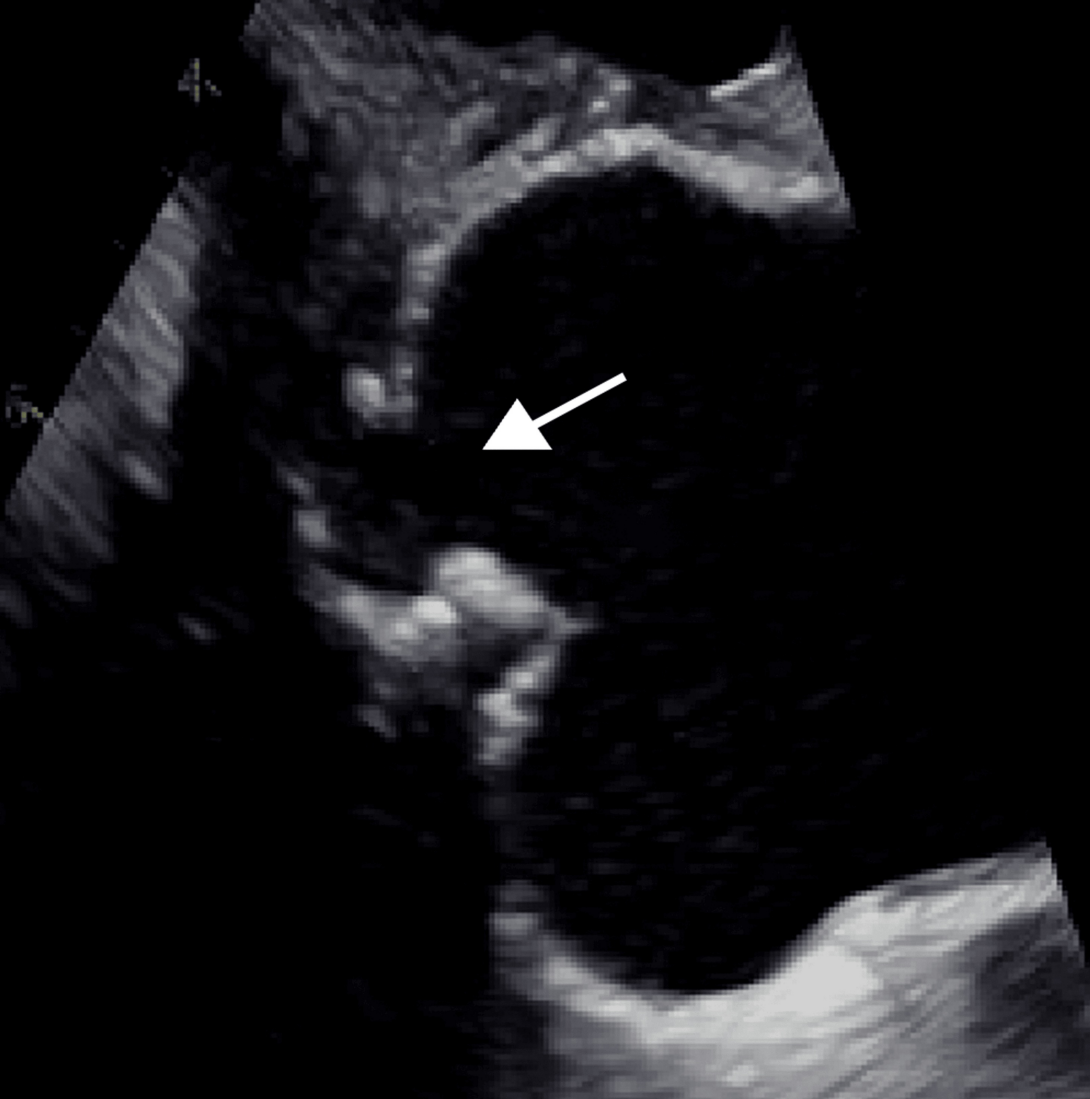
Cardiac MRI showing suspected aortic root aneurysmal outpouching/abscess The arrow shows the outpouching (abscess) on the non-coronary aortic valve cusp

The patient was diagnosed with MSSA bacteremia likely secondary to his dialysis catheter. The catheter was promptly removed, and intravenous nafcillin was initiated. Despite appropriate antimicrobial therapy, he remained persistently bacteremic with ongoing fevers. Over the next 48 hours, the patient developed new-onset first-degree atrioventricular (AV) block (Figure 1), which progressed to Mobitz type II AV block on serial ECGs. A temporary transvenous pacemaker was placed due to the risk of complete heart block. Transesophageal echocardiography (TEE) was performed, which demonstrated a large mobile vegetation at the base of the non-coronary cusp of the aortic valve, with aneurysmal calcifications extending toward the right atrium. These findings were concerning for a developing aortic root abscess. Cardiac MRI showed aortic root aneurysmal outpouching concerning for abscess (Figure 2). 

Surgical evaluation determined that the patient was not a candidate for urgent valve surgery due to ESRD, coagulopathy, thrombocytopenia, and liver dysfunction. Despite continued IV antibiotic therapy and hemodynamic support, the patient deteriorated clinically. He developed signs of cardiogenic shock, with escalating vasopressor requirements and end-organ hypoperfusion. Repeat echocardiography showed worsening perivalvular involvement. On hospital day 15, the patient experienced a pulseless electrical activity cardiac arrest and died despite resuscitation efforts. 

## Discussion

Aortic root abscess is a severe and life-threatening complication of IE, particularly when involving the aortic valve annulus. The development of conduction abnormalities such as AV block in the setting of IE is a well-established clinical indicator of perivalvular extension and is frequently associated with intracardiac abscess formation [1,5,8]. Our case highlights this progression: an initially stable dialysis-dependent patient with MSSA bacteremia and normal sinus rhythm who developed aortic root abscess with high-grade AV block and ultimately succumbed to septic and cardiogenic shock. 

Intracardiac abscesses are reported in 30-40% of patients with IE, most commonly involving the aortic valve, especially when infection is caused by virulent organisms such as Staphylococcus aureus [1,6]. The annular involvement is particularly dangerous due to the proximity of the conduction system; the AV node and the His bundle lie near the membranous septum adjacent to the non-coronary and right coronary cusps of the aortic valve. Infection or inflammation in this area can result in conduction delays, as seen in our patient who progressed from a prolonged PR interval to Mobitz type II AV block [3,7]. 

Dialysis-dependent patients are at markedly increased risk for bloodstream infections and subsequent endocarditis, with S. aureus being the most common causative pathogen. Studies have shown that bacteremia in this population carries a high risk of complications, including endocardial involvement and embolic events [10,11]. Our patient’s initial bacteremia likely originated from the tunneled dialysis catheter, despite no overt signs of local infection, consistent with findings that S. aureus bacteremia can be hematogenously seeded even in the absence of localized inflammation [11]. 

Echocardiography remains the primary diagnostic tool in the evaluation of suspected endocarditis. While transthoracic echocardiography has limited sensitivity in detecting abscesses, TEE offers superior imaging resolution, particularly in evaluating perivalvular extension, prosthetic valve involvement, and abscess cavities [12]. In our patient, TEE was pivotal in identifying the vegetation and suspected abscess, prompting further escalation of care. 

The presence of an aortic root abscess in IE usually mandates surgical intervention. Early surgical debridement and valve replacement have been shown to improve survival, particularly in patients with perivalvular complications, persistent bacteremia, or conduction abnormalities [4,5]. However, surgery in hemodynamically unstable patients with significant comorbidities may not be feasible, as in this case. The patient’s liver dysfunction, thrombocytopenia, and ongoing sepsis precluded emergent surgical repair, consistent with literature highlighting the poor prognosis in patients deemed non-operative candidates [4,13]. 

This case also reaffirms the prognostic value of new conduction abnormalities in IE. As highlighted by Brown et al. and the European Society of Cardiology (ESC) guidelines, AV block is a clinical red flag for perivalvular extension and should prompt urgent imaging and surgical consultation [1,8]. Unfortunately, despite guideline-directed therapy, the lack of surgical intervention resulted in a fatal outcome in this patient. 

## Conclusions

This case underscores the importance of early recognition of perivalvular abscess in patients with IE. This case highlights the critical importance of early recognition and aggressive evaluation of intracardiac complications in patients with IE, particularly those at increased risk such as individuals on chronic hemodialysis. The development of new conduction abnormalities, even in the absence of overt cardiac symptoms, should immediately raise suspicion for perivalvular extension and possible aortic root abscess. Timely imaging with TEE and prompt multidisciplinary evaluation are essential to guide both medical and surgical management. Unfortunately, patients with significant comorbidities may not be eligible for surgical intervention, which substantially worsens prognosis. Clinicians must maintain a high index of suspicion and act decisively, as delays in recognition or limitations in treatment options can result in rapid clinical decline and mortality.
